# Supplementary material for: Bacterial Persister-Cells and Spores in the Food Chain: Their Potential Inactivation by Antimicrobial Peptides (AMPs)
Source: Int J Mol Sci. 2020 Nov 27;21(23):8967. doi: 10.3390/ijms21238967 (PMC7731242; doi:10.3390/ijms21238967)
Supplement: Supplementary file 1 [file ijms-21-08967-s001.pdf]

Table S1. Additional information of antimicrobial peptides mentioned in this review.

Name	Origin	Structure	Amino Acid Sequence	Targets	Ref.
PGLa	Skin of *Xenopus laevis*	Coil/ α-helical	GMASKAGAIAGKIAKV ALKAL-NH_2	Membrane	[1]
Magainins-1	Skin of *Xenopus laevis*	α-helical	GIGKFLHSAGKFGKAF VGEIMKS	Membrane	[2]
Magainins-2	Skin of *Xenopus laevis*	α-helical	GIGKFLHSAKKFGKAF VGEIMNS	Membrane	[2]
alamethicin	*Trichoderma viride*	α-helical	Ac-UPUAUAQUVU GLUPVUUEQ-Phol	Membrane	[3]
caerin 1.1	Skin of Australian tree frogs	α-helical	GLLSVLGSVAKHVLPH VVPVIAEHL-NH2	Membrane	[4]
LL-37	Human cathelicidin	α-helical	LLGDFFRKSKEKIGKEF KRIVQRIKDFLRNLVPR TES	Membrane	[5] [6]
Buforin II	Histone H2A	α-helical	TRSSRAGLQFPVGRVH RLLRK	Nucleic acids	[7]
Indolicidin	Bovine neutrophils	extended	ILPWKWPWWPWRR-NH_2	Duplex DNA	[8]
CP10A	Derivative of indolicidin	helical	ILAWKWAWWAWRR-NH_2	Membrane, Macromolecular Synthesis	[8]
Microcin 25	*Escherichia coli*	lariat protoknot	GGAGHVPEYFVGIGTPI SFYG	RNA polymerase, respiratory chain	[9,10]
eNAP-2	Equine neutrophils	polycationic	EVERKHPLGGSRPGRC PTVPPGTFGHCACLCT GDASEPKGQKCCSN	Microbial serine proteases	[11,12]
Nisin	*Lactococcus lactis*	Polycyclic, lantibiotic	IOAIULAAPGAKAGAL MGANMKAAAANASI HVUK	Lipid II	[13]
SAAP-148	Derived from LL-37	α-helical	LKRVWKRVFKLLKRY WRQLKKPVR	Membrane	[14]
TC-19	Derived from thrombocidin-1	α-helical	LRCMCIKWWSGKHPK	Membrane	[15]
TC-84	Derived from thrombocidin-1	α-helical	LRAMCIKWWSGKHPK	Membrane	[15]
BP2	Designed based on human BPI	α-helical	GKWKLFKKAFKKFLKI LAC	Membrane	[15]
lacticin 3147	*Lactococcus lactis*	lantibiotic	HSDGIFTDSYSRYRKQ MAVKKYLAAVL-NH_2	Lipid II	[16]
Leucocin A	*Leuconostoc gelidum*	β-sheet/ α-helical	KYYGNGVHCTKSGCS VNWGEAFSAGVHRLA NGGNGFW	Membrane	[17]

BPI: bactericidal permeability increasing protein.

1. Wieprecht, T.; Apostolov, O.; Beyermann, M.; Seelig, J. Membrane Binding and Pore Formation of the Antibacterial Peptide PGLa: Thermodynamic and Mechanistic Aspects. *Biochemistry* **2000**, *39*, 442–452.

2. Zasloff, M. Magainins, a class of antimicrobial peptides from Xenopus skin: isolation, characterization of two active forms, and partial cDNA sequence of a precursor. *Proc. Natl. Acad. Sci. U. S. A.* **1987**, *84*, 5449–5453.

3. Mihajlovic, M.; Lazaridis, T. Antimicrobial peptides in toroidal and cylindrical pores. *Biochim. Biophys. Acta* **2010**, *1798*, 1485–1493.

4. Laadhari, M.; Arnold, A.A.; Gravel, A.E.; Separovic, F.; Marcotte, I. Interaction of the antimicrobial peptides caerin 1.1 and aurein 1.2 with intact bacteria by 2H solid-state NMR. *Biochim. Biophys. Acta - Biomembr.* **2016**, *1858*, 2959–2964.

5. Turner, J.; Cho, Y.; Dinh, N.N.; Waring, A.J.; Lehrer, R.I. Activities of LL-37, a cathelin-associated antimicrobial peptide of human neutrophils. *Antimicrob. Agents Chemother.* **1998**, *42*, 2206–2214.

6. Kang, H.K.; Kim, C.; Seo, C.H.; Park, Y. The therapeutic applications of antimicrobial peptides (AMPs): a patent review. *J. Microbiol.* **2017**, *55*, 1–12.

7. Park, C.B.; Yi, K.-S.; Matsuzaki, K.; Kim, M.S.; Kim, S.C. Structure–activity analysis of buforin II, a histone H2A-derived antimicrobial peptide: The proline hinge is responsible for the cell-penetrating ability of buforin II. *Proc. Natl. Acad. Sci.* **2000**, *97*, 8245 LP – 8250.

8. Friedrich, C.L.; Rozek, A.; Patrzykat, A.; Hancock, R.E.W. Structure and Mechanism of Action of an Indolicidin Peptide Derivative with Improved Activity against Gram-positive Bacteria. *J. Biol. Chem.* **2001**, *276*, 24015–24022.

9. Mukhopadhyay, J.; Sineva, E.; Knight, J.; Levy, R.M.; Ebright, R.H. Antibacterial Peptide Microcin J25 Inhibits Transcription by Binding within and Obstructing the RNA Polymerase Secondary Channel. *Mol. Cell* **2004**, *14*, 739–751.

10. Galván, A.E.; Chalón, M.C.; Ríos Colombo, N.S.; Schurig-Briccio, L.A.; Sosa-Padilla, B.; Gennis, R.B.; Bellomio, A. Microcin J25 inhibits ubiquinol oxidase activity of purified cytochrome bd-I from Escherichia coli. *Biochimie* **2019**, *160*, 141–147.

11. Couto, M.A.; Harwig, S.S.; Cullor, J.S.; Hughes, J.P.; Lehrer, R.I. eNAP-2, a novel cysteine-rich bactericidal peptide from equine leukocytes. *Infect. Immun.* **1992**, *60*, 5042–5047.

12. Couto, M.A.; Harwig, S.S.; Lehrer, R.I. Selective inhibition of microbial serine proteases by eNAP-2, an antimicrobial peptide from equine neutrophils. *Infect. Immun.* **1993**, *61*, 2991–2994.

13. Le, C.-F.; Fang, C.-M.; Sekaran, S.D. Intracellular Targeting Mechanisms by Antimicrobial Peptides. *Antimicrob. Agents Chemother.* **2017**, *61*, e02340-16.

14. De Breij, A.; Riool, M.; Cordfunke, R.A.; Malanovic, N.; De Boer, L.; Koning, R.I.; Ravensbergen, E.; Franken, M.; Van Der Heijde, T.; Boekema, B.K.; et al. The antimicrobial peptide SAAP-148 combats drug-resistant bacteria and biofilms. *Sci. Transl. Med.* **2018**, *10*.

15. Omardien, S.; Drijfhout, J.W.; van Veen, H.; Schachtschabel, S.; Riool, M.; Hamoen, L.W.; Brul, S.; Zaat, S.A.J. Synthetic antimicrobial peptides delocalize membrane bound proteins thereby inducing a cell envelope stress response. *Biochim. Biophys. Acta - Biomembr.* **2018**, *1860*, 2416–2427.

16. Iancu, C.; Grainger, A.; Field, D.; Cotter, P.D.; Hill, C.; Ross, R.P. Comparison of the Potency of the Lipid II Targeting Antimicrobials Nisin, Lacticin 3147 and Vancomycin Against Gram-Positive Bacteria. *Probiotics Antimicrob. Proteins* **2012**, *4*, 108–115.

17. Balandin, S. V.; Sheremeteva, E. V.; Ovchinnikova, T. V. Pediocin-Like Antimicrobial Peptides of Bacteria. *Biochem.* **2019**, *84*, 464–478 ISBN 0006297919.
